# Resveratrol Prevents Breast Cancer Metastasis by Inhibiting Wnt/β-Catenin Pathway-Mediated Epithelial–Mesenchymal Transition

**DOI:** 10.3390/ph19010041

**Published:** 2025-12-23

**Authors:** Xue Fang, En Ma, Runshu Wang, Jingwei Zhang, Yirong Tang, Jinxiao Chen, Weidong Zhu, Da Wo, Dan-ni Ren

**Affiliations:** Academy of Integrative Medicine, College of Integrative Medicine, Fujian Key Laboratory of Integrative Medicine on Geriatric, Fujian University of Traditional Chinese Medicine, Fuzhou 350122, Fujian, China; 15705961231@163.com (X.F.); 18217497624@163.com (E.M.); runshu_wang199@163.com (R.W.); 15803856395@163.com (J.Z.); trong750@163.com (Y.T.); shangtang002002@163.com (J.C.); wzhu@tongji.edu.cn (W.Z.)

**Keywords:** resveratrol, lung metastasis, breast cancer, Wnt/β-catenin signaling pathway, epithelial–mesenchymal transition

## Abstract

**Background:** Breast cancer is the most prevalent cancer in women, and metastatic breast cancer remains a major cause of cancer-related deaths. Resveratrol (RSV) is a natural compound found in various plants and is known to exhibit various anti-cancer effects. The present study aims to investigate the therapeutic effects and mechanisms of RSV in inhibiting breast cancer metastasis in a murine model of 4T1 breast tumor that shares close molecular features with human triple negative breast cancer. **Methods**: Murine breast cancer 4T1 cells were used to examine the effects of RSV on breast cancer metastasis and epithelial–mesenchymal transition (EMT). In vitro cell proliferation and Transwell migration assays and in vivo 4T1 tumor transplantation models were established in female Balb/c mice to determine the anti-metastatic effects of RSV and its mechanism of action. **Results**: RSV significantly inhibited 4T1 tumor cell migration and significantly decreased expression levels of EMT markers Snail and Vimentin, as well as the nuclear translocation of β-catenin both in vitro and in vivo. Knockdown of β-catenin similarly reduced the expression levels of EMT markers. RSV significantly decreased the number of lung metastases in 4T1-implanted mice by inhibiting Wnt/β-catenin signaling pathway activation. RSV (150 mg/kg/day) reduced the number of visible tumor metastatic nodules and the histological count of metastatic lung carcinomas by 51.82% and 62.58%, respectively, compared to vehicle administration. **Conclusions**: Our study provides important new mechanistic insight into the strong anti-cancer effects of RSV in inhibiting 4T1 breast cancer metastasis by preventing Wnt/β-catenin signaling pathway-mediated epithelial–mesenchymal transition. These findings suggest the therapeutic potential of RSV as a promising drug in the treatment of metastatic breast cancer.

## 1. Introduction

Breast cancer is the most prevalent malignancy in women and one of the leading causes of cancer-related deaths worldwide [1]. Although the prognosis of patients with early-stage breast cancer has improved in recent years, the treatment of advanced metastatic breast cancer remains a key challenge [2]. Therefore, understanding the key molecular mechanisms involved during breast cancer metastasis, as well as the development of effective targeted intervention strategies are essential in improving patient survival.

Epithelial–mesenchymal transition (EMT) plays a central driving role in the metastatic progression of breast cancer and facilitating metastatic dissemination by remodeling the invasive and migratory capacity of cancer cells [3]. Numerous EMT transcription factors have been shown to play key roles in directing migration of MCF-7 human breast cancer cells to the skeletal microenvironment [4], as well as accelerating metastatic progression in triple-negative breast cancer [5]. Upregulation of mesenchymal marker Vimentin by the transcription factor Snail is also a key factor in the regulation of EMT. In contrast, inhibition of EMT robustly suppresses breast cancer metastasis [6]. These findings reveal the critical role of EMT in breast cancer metastasis and highlight its potential as an important therapeutic target.

The Wnt/β-catenin signaling pathway is a key pathway in the regulation of EMT, whereby nuclear translocation of β-catenin activates the transcriptional programs of downstream EMT-related genes [7,8]. Several studies have shown that targeting the Wnt/β-catenin signaling pathway can effectively inhibit EMT and breast cancer metastasis [9,10]. Therefore, targeting the Wnt/β-catenin pathway in the regulation of tumor cell EMT is an important strategy in inhibiting breast cancer metastasis.

Resveratrol (RSV) is a natural active compound commonly found in various plants including *Polygonum cuspidatum* and grapes [11], and it has been found to possess various pharmacological activities such as antioxidant, anti-inflammatory, anti-tumor, and immunomodulatory activities [12,13]. The anti-cancer effects of resveratrol are due to its low toxicity and selective cytotoxicity against cancer cells [14]. Studies have shown that resveratrol inhibits the proliferation of breast cancer stem cell-like cells and induces autophagy by inhibiting the Wnt/β-catenin signaling pathway, thereby inhibiting tumor growth [15]. Resveratrol inhibits EMT induced by the epidermal growth factor in MCF-7 cells through regulation of the ERK signaling pathway [16].

In this study, we utilized the 4T1 murine mammary gland tumor model, whose phenotypic characteristics and molecular features are comparable to human triple-negative breast cancer (TNBC), including its highly aggressive and poorly immunogenic nature, as well as spontaneous metastatic progression [17,18]. 4T1 cells have been suggested to possess activated Wnt signaling due to the upregulation of multiple Wnt ligands [17]. Although numerous studies have reported on the anti-cancer effects of resveratrol, our study fills the gap in elucidating the roles and mechanisms of resveratrol in suppressing breast tumor metastasis via in vivo demonstration of nuclear β-catenin inhibition. Further, our study aimed to clarify the anti-metastatic effects of RSV via targeted inhibition of the Snail/Vimentin axis and suppression of tumor epithelial–mesenchymal transition. This study was performed in accordance with the MDAR and ARRIVE reporting checklists.

## 2. Results

### 2.1. RSV Inhibits Proliferation and Migration of 4T1 Breast Cancer Cells

**Figure 1:**
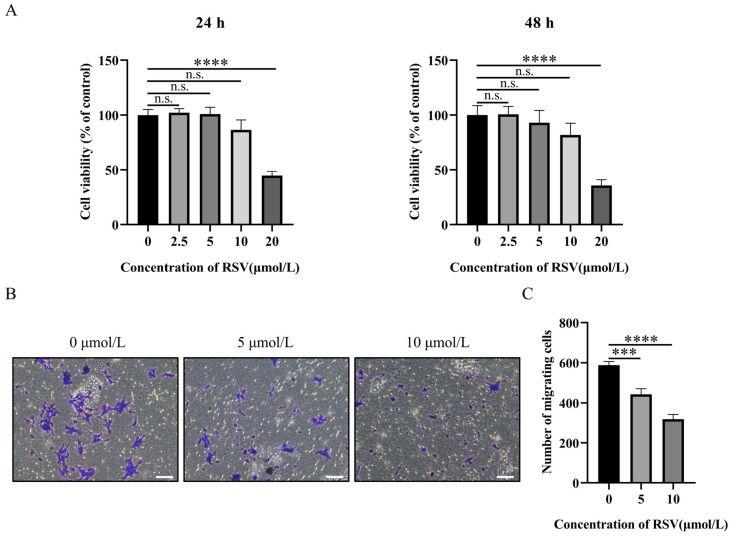
Effect of resveratrol (RSV) on proliferation and migration of 4T1 cells. (**A**) CCK8 assay of cell viability in 4T1 cells treated with different concentrations of RSV for 24 h (left) and 48 h (right). (**B**,**C**) Representative images (**B**) and quantification (**C**) of migrating cells in purple treated with RSV for 20 h (100× magnification). Scale bar 100 μm. Data are presented as mean ± SD. One-way ANOVA, Tukey’s post hoc test, not significant (n.s.), *** *p* < 0.001, **** *p* < 0.0001, *n* = 3 independent experiments.

We first examined the potential effects of RSV in inhibiting proliferation and migration of 4T1 breast cancer cells. CCK-8 and lactate dehydrogenase (LDH) assays showed that 4T1 cells treated with 2.5–10 μmol/L RSV for 24 or 48 h exhibited no significant changes in cell viability (Figure 1A, Supplementary Figure S1A). However, cell viability was significantly reduced in the 20 μmol/L treatment group, suggesting that RSV exhibits cytotoxicity at this concentration (Figure 1A, Supplementary Figure S1A). Therefore, we utilized RSV concentrations of 5 μmol/L and 10 μmol/L for subsequent experiments. Phase-contrast images showed that RSV decreased the 4T1 cell density (Supplementary Figure S1B). We further examined the migration potential of 4T1 cells via a Transwell migration assay, which showed that RSV treatment for 20 h significantly inhibited the migration ability of 4T1 cells in a dose-dependent manner (Figure 1B,C).

### 2.2. RSV Prevents Breast Cancer Cell Metastasis by Inhibiting EMT via Regulation of Wnt/β-Catenin Signaling Pathway

**Figure 2:**
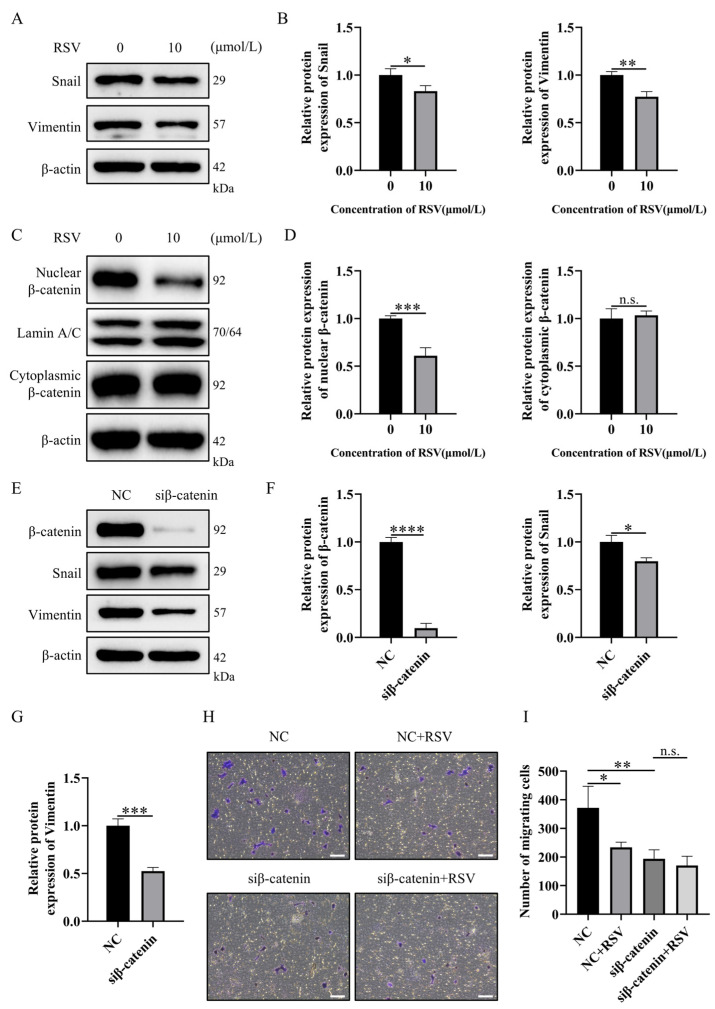
Resveratrol (RSV) prevents breast cancer cell metastasis by inhibiting epithelial–mesenchymal transition (EMT) mediated by the Wnt/β-catenin signaling pathway. (**A**,**B**) Representative immunoblots (**A**) and quantification (**B**) of Snail and Vimentin expression in 4T1 cells treated with RSV for 48 h. (**C**,**D**) Representative immunoblots (**C**) and quantification (**D**) of nuclear or cytoplasmic β-catenin expression in 4T1 cells treated with RSV for 48 h, *n* = 4 independent experiments. (**E**–**G**) Representative immunoblots (**E**) and quantification (**F**,**G**) of β-catenin, Snail, and Vimentin expression in 4T1 cells treated with β-catenin knockdown for 48 h. (**H**,**I**) Representative images (**H**) and quantitative analysis (**I**) of migrating cells in purple treated with RSV for 20 h after 48 h of β-catenin knockdown or no knockdown in 4T1 cells (100× magnification). NC, negative control. Scale bar 100 μm. Data are presented as mean ± SD. Student’s *t*-test or one-way ANOVA, Tukey’s post hoc test, not significant (n.s.), * *p* < 0.05, ** *p* < 0.01, *** *p* < 0.001, **** *p* < 0.0001, *n* = 3 independent experiments.

Next, we examined the role and mechanism of RSV in preventing epithelial–mesenchymal transition in 4T1 breast cancer cells. Western blot analysis showed that the expression levels of EMT core transcription factor Snail, mesenchymal marker Vimentin, and classical EMT marker N-cadherin were significantly reduced in 4T1 cells treated with 10 μmol/L RSV for 48 h compared with the control group (Figure 2A,B, Supplementary Figure S2A,B). In addition, nuclear accumulation of β-catenin as well as Wnt target genes c-Myc and Rnf43 was similarly reduced in RSV-treated cells, while the cytoplasmic β-catenin level remained unchanged (Figure 2C,D, Supplementary Figure S3A,B). To further investigate the role of the Wnt/β-catenin pathway in the regulation of EMT in 4T1 cells, we used RNA interference to silence the expression of β-catenin, which resulted in significantly decreased levels of Snail and Vimentin compared to the control group (Figure 2E–G). In order to verify whether RSV regulates the EMT process by targeting the Wnt/β-catenin pathway, we further treated 4T1 cells with RSV following siRNA knockdown of β-catenin. Knockdown efficiency was confirmed by both WB and real-time PCR (Figure 2F, Supplementary Figure S4). A Transwell migration assay showed that knockdown of β-catenin for 48 h significantly inhibited 4T1 cell migration, which was similar to the inhibitory effect of RSV alone (Figure 2H,I). However, there was no additive effect of RSV treatment on 4T1 cells following siRNA knockdown of β-catenin, suggesting that RSV blocks the EMT-mediated cancer cell migration process by inhibiting the Wnt/β-catenin pathway.

### 2.3. RSV Inhibits Lung Metastasis in 4T1 Breast Cancer Mice

**Figure 3:**
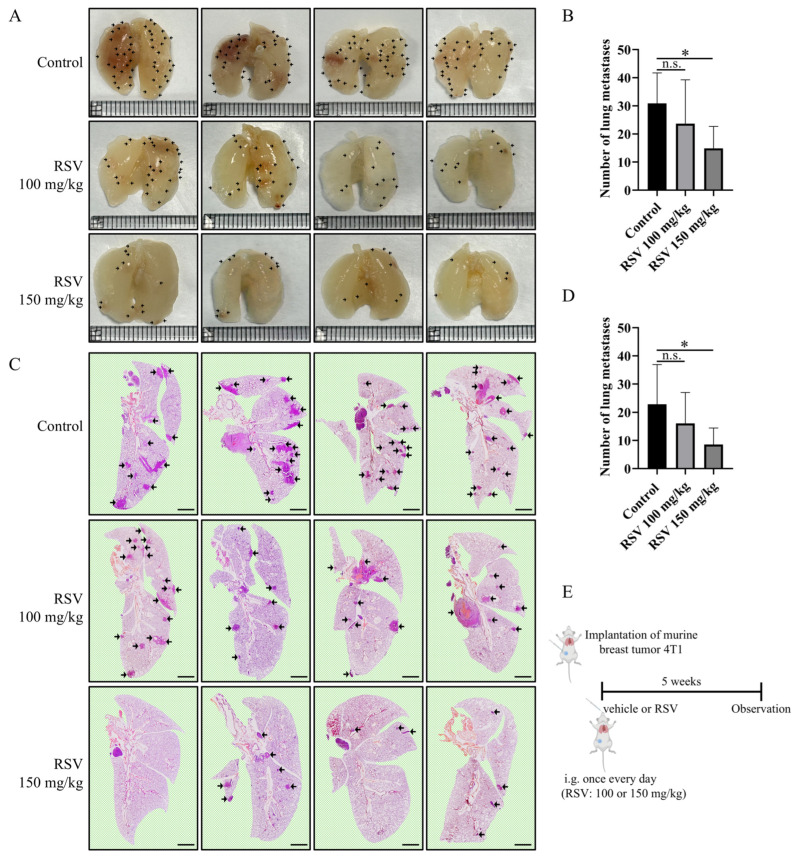
Resveratrol (RSV) inhibits lung metastasis of breast cancer in mice. Mice were administered daily with RSV (100 or 150 mg/kg) or an equal volume of vehicle for 5 weeks. (**A**) Intact lung showing lung metastases in 4T1 transplanted tumor mice; each small square represents 1 mm; black arrows highlight visible tumor metastatic nodules. (**B**) Quantification of lung metastases in mice. (**C**) H&E staining showing representative images of metastatic foci of right lungs in mice, 25× magnification; scale bar, 2 mm; black arrows highlight nodular aggregates of atypical cells consistent with metastatic carcinoma. (**D**) Quantification of intact lung metastases in mice. (**E**) Schematic model of 4T1 mouse tumor implantation. Following implantation with 4T1 breast tumor cells, RSV (100 or 150 mg/kg) or an equal volume of vehicle was administered daily via intragastric (i.g.) administration for 5 weeks. Data are presented as mean ± SD. One-way ANOVA, Tukey’s post hoc test, not significant (n.s.), * *p* < 0.05, *n* = 9 mice in each group.

To assess the inhibitory effect of RSV on lung metastasis in vivo, we performed in situ transplantation of 4T1 cells in mice and counted the number of visible lung metastatic tumor nodules, as well as histologically examined lung metastases after 5 weeks. Although there was no difference in primary tumor weight, the tumor-free weight of mice administered RSV (150 mg/kg) was significantly higher than the control group (Supplementary Figure S5A–C). The low-dose RSV group (100 mg/kg) had a decreased number of visible lung tumor metastatic nodules (23.67 ± 15.59) versus the vehicle (30.89 ± 10.83), albeit statistically insignificant, while the high-dose RSV group (150 mg/kg) significantly reduced the formation of lung metastases (14.89 ± 7.79) (Figure 3A,B). H&E histological analysis of lung sections exhibited a similar trend, where the number of nodular aggregates of atypical cells consistent with metastatic carcinoma decreased in the low-dose RSV group (16.11 ± 10.97) versus the vehicle (22.89 ± 14.07) and significantly decreased in the high-dose RSV group (8.56 ± 5.88) (Figure 3C,D). A schematic of the timeline for the in vivo model is shown in Figure 3E. Taken together, these results demonstrate the robust effect of RSV treatment in preventing the metastasis of 4T1 breast cancer in mice.

### 2.4. RSV Downregulates Tumor EMT by Inhibiting β-Catenin Nuclear Translocation

**Figure 4:**
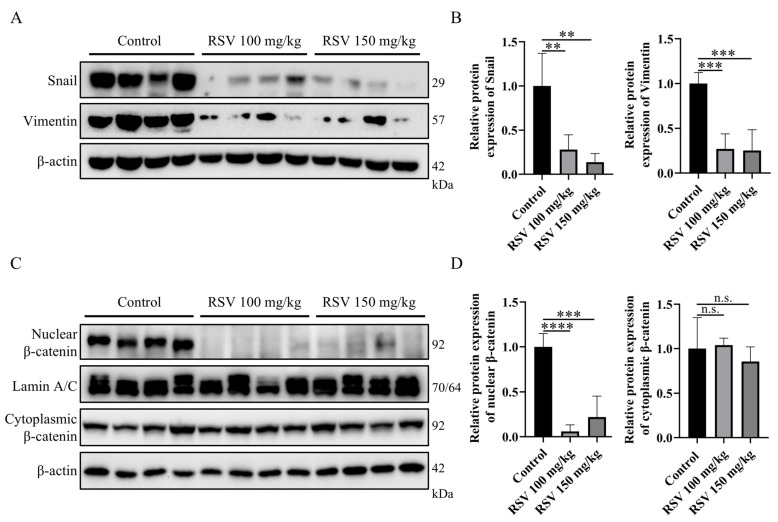
Resveratrol (RSV) downregulates tumor tissue EMT by inhibiting β-catenin nuclear translocation. Mice were administered daily with RSV (100 or 150 mg/kg) or an equal volume of vehicle for 5 weeks. (**A**,**B**) Representative immunoblots (**A**) and quantification (**B**) of Snail and Vimentin expression in tumor tissues of mice administrated with the vehicle or RSV for 5 weeks. (**C**,**D**) Representative immunoblots (**C**) and quantification (**D**) of nuclear or cytoplasmic β-catenin expression in tumor tissues Data are presented as mean ± SD. One-way ANOVA, Tukey’s post hoc test. not significant (n.s.), ** *p* < 0.01, *** *p* < 0.001, **** *p* < 0.0001, *n* = 4.

We further examined the expression levels of Snail and Vimentin in the tumor tissues of 4T1-implanted mice after 5 weeks of RSV intervention. Western blot analysis showed that RSV treatment (100 mg/kg and 150 mg/kg) significantly inhibited the protein expression levels of EMT marker Snail, mesenchymal marker Vimentin, and classical EMT marker N-cadherin in the tumor tissue (Figure 4A,B, Supplementary Figure S2C,D). Furthermore, the levels of nuclear β-catenin as well as Wnt target genes c-Myc and Rnf43 in the tumor tissue were also significantly reduced in RSV-administered mice, while the cytoplasmic β-catenin level remained unchanged (Figure 4C,D, Supplementary Figure S3C–F). Taken together, these results demonstrate that resveratrol prevents the epithelial–mesenchymal transition of 4T1 tumors via inhibition of the Wnt/β-catenin pathway.

## 3. Discussion

One of the main outcomes of metastatic breast cancer is the formation of lung metastases, which directly impair the respiratory function and lead to a worsened patient prognosis [19]. Hence, intervention strategies targeting the metastatic potential of breast tumors are crucial. Epithelial–mesenchymal transition (EMT) is a key process driving breast cancer metastasis, which confers to tumor cells the ability to invade and metastasize [20]. Notably, the transcription factor Snail and its downstream effector Vimentin are core factors regulating EMT in tumors. Clinical studies have shown that elevated expression of Snail is significantly associated with a higher pathological grade of breast cancer, coupled with an increased risk of distant metastasis and a poor patient prognosis [21,22]. Vimentin is a key downstream effector molecule of Snail/Slug [23] that has been shown to be positively correlated with the metastatic progression of breast cancer [24,25,26,27]. Numerous studies have demonstrated that targeted inhibition of Snail can effectively suppress EMT and reduce metastasis. For example, thujaplicin has been shown to inhibit EMT and lung metastasis by downregulating Snail expression [28], while omeprazole inhibits tumor invasion and metastasis by promoting degradation of Snail protein [29]. Therefore, targeted inhibition of the Snail/Vimentin axis may be an important strategy in inhibiting EMT and breast cancer metastasis.

Resveratrol is the main active ingredient of various plants, including the traditional Chinese medicine *Polygonum cuspidatum*, and has been shown to exhibit strong anti-tumor effects. Previous studies have reported that RSV can inhibit breast cancer metastasis by regulating EMT [30]. Our current study extends previous observations by demonstrating that RSV inhibits tumor cell EMT via targeted inhibition of the Snail/Vimentin axis, in both the in vivo model of 4T1 breast tumor metastasis as well as in vitro inhibition of cancer cell migration. These results demonstrate that the anti-metastatic effects of RSV are closely related to the inhibition of the Snail/Vimentin-mediated EMT process.

Our current study showed that the regulation of Snail/Vimentin by RSV was via inhibition of the Wnt/β-catenin signaling pathway, one of the key pathways involved in the regulation of the EMT process [8]. The Wnt/β-catenin signaling pathway can activate EMT by either inducing Axin2 expression and stabilizing the Snail protein [31], or through EMT-related transcription factors by inhibiting GSK-3β-mediated degradation of β-catenin and Snail [32]. Notably, Snail is not only a key effector molecule of this pathway but also its direct transcriptional target [31,33]. In our study, we found that resveratrol administration significantly decreased nuclear levels of β-catenin, and the effect was much more evident in the tumor tissues of mice than in vitro treatment in 4T1 cells. We postulate that because mice were administered daily with resveratrol for 35 days, whereas cells were only treated once prior to collection after 48 h, this led to an increase in the inhibitive capacity and availability of resveratrol.

There are a few limitations to our study. Firstly, the clinical therapeutic potential of resveratrol is limited by its pharmacokinetic issues, including rapid metabolism and low bioavailability following oral administration [34,35]. Despite this limitation, our study showed that daily intragastric administration of resveratrol at a 150 mg/kg dosage was sufficient for inhibiting cancer metastasis and EMT. Secondly, we used only 4T1 cells in both our in vivo and in vitro studies, and hence further examinations with separate cell lines would be required to better verify the effects of resveratrol in breast cancer. Nevertheless, because 4T1 cells share substantial molecular features with human triple-negative breast cancer (TNBC), including its highly aggressive, metastatic, and poor immunogenic nature [17,18], the observed anti-metastatic effect of resveratrol suggests its therapeutic potential in human breast cancer, in particular TNBC.

Taken together, our study found that RSV treatment effectively inhibited the nuclear translocation of β-catenin, thereby preventing Wnt/β-catenin signaling activation. Importantly, our β-catenin knockdown experiments confirmed that the Wnt/β-catenin pathway plays a central role in regulating Snail/Vimentin expression and cancer cell migration. In particular, RSV treatment had no additive effect in cells with β-catenin knockdown, which supported our conclusion that RSV prevents EMT of breast tumors by inhibiting the Wnt/β-catenin pathway.

## 4. Materials and Methods

### 4.1. Preparation of Resveratrol

Resveratrol (purity 99.94%) was purchased from MedChemExpress (MCE, South Brunswick, NJ, USA). For in vitro experiments, 10 mmol/L stock solution of RSV was dissolved in dimethyl sulfoxide (DMSO, Sangon Biotech, Shanghai, China). For in vivo experiments, 200 mg/mL stock solution of RSV was prepared from a mixture of 25% polyethylene glycol 400 (PEG400, Sigma Aldrich, St. Louis, MO, USA) and 75% DMSO, and then the stock solution was diluted to the target concentration with a mixture of 20% PEG400 and 80% PBS before use. Both solvents are FDA-approved and exhibited no toxicity during long-term in vivo administration [36]. Resveratrol dosages of 100 and 150 mg/kg were selected for in vivo metastasis experiments, as previously described [37].

### 4.2. Cell Culture

The mouse mammary carcinoma cell line 4T1 was obtained from the American Type Culture Collection (ATCC). We cultured 4T1 cells in high-sugar DMEM (containing 10% fetal bovine serum, 1% penicillin–streptomycin) in a 37 °C incubator supplemented with 5% carbon dioxide.

### 4.3. Cytotoxicity Assays CCK-8 and LDH

We seeded 4T1 cells in a 96-well plate (8000–10,000 cells/well in 100 μL complete medium) and incubated them overnight (37 °C, 5% CO_2_) for attachment. After treatment with RSV for 24 h and 48 h, 10 μL of CCK-8 reagent (Apex Bio, Houston, TX, USA) or 100 μL of LDH reagent (Beyotime, Shanghai, China) was added to each well, and subsequent absorbances were measured at 450 nm.

### 4.4. Transwell Experiment

We divided 4T1 cells into three groups: control, 5 μmol/L RSV, and 10 μmol/L RSV cultured in Transwell chambers with 750 μL of cell culture medium containing 0.5% FBS added to the lower chamber and 500 μL of serum-free medium to the upper chamber. Cells (40,000/well) were seeded into the upper chamber and incubated for 20 h. Subsequently, the cells were fixed with 4% paraformaldehyde for 15 min and stained with crystal violet for 30 min. After wiping off the cells in the upper chamber with a cotton swab, a total of seven fields of view (both peripheral and central regions) were captured per chamber to ensure a comprehensive coverage, and the mean numbers of migrating cells in each group were quantified.

### 4.5. Western Blot

Total proteins were extracted using NP-40 (Beyotime, Shanghai, China) or RIPA (Beyotime, China), and nuclear proteins were extracted using the Nuclear Protein Kit (Sangon Biotech, Shanghai, China). Protein concentrations were detected by the BCA method, and then equal amounts of samples were separated by SDS-PAGE and transferred onto PVDF membranes. Membranes were blocked with 5% non-fat milk for 2 h and incubated with primary antibodies at 4 °C overnight. Subsequently, membranes were incubated with the corresponding secondary antibody for 1.5 h and detected via chemiluminescence. The following antibodies were used: Snail (cat# 3879S), Vimentin (cat# 3932), β-catenin (cat# 8480), and c-Myc (cat# 9402S) from Cell Signaling Technology, Danvers, MA, USA; β-actin (cat# 60008-1-Ig), Lamin A/C (cat# 10298-1-AP), GAPDH (cat# 60004-1-Ig), and RNF43 (cat# 86740-1-RR) from Proteintech, Chicago, IL, USA; and N-cadherin (cat# ab76011) from Abcam, Cambridge, UK. We also used Goat anti-Rabbit-IgG (cat# L3012) and Goat anti-Mouse-IgG (cat# L3032) from SAB, USA. Western blot band intensities were quantified using Image J software (version 1.52i, National Institutes of Health, USA) and normalized to the corresponding loading controls.

### 4.6. SiRNA Transfection Assay

SiRNA oligo (cat# s63417, Thermo Fisher Scientific, Waltham, MA, USA) and lipofectamine RNAiMAX (cat# 13778150, Thermo Fisher Scientific, USA) were added in Opti-MEM (cat# 11058021, Thermo Fisher Scientific, USA), and the mixture was allowed to stand for 15 min to form a transfection complex. Subsequently, the transfection complex was gently added to the cell culture medium and incubated for 48 h.

### 4.7. Murine Breast Tumor Model

Female Balb/c mice (6–8 weeks old, 20–23 g) were obtained from Shanghai SLAC Laboratory Animal Co., Ltd. (Shanghai, China) and housed in an SPF-grade temperature-controlled room at 24 °C with a 12 h light–dark cycle, with tap water and rodent chow available ad libitum. Twenty-seven mice were randomly divided into three groups: control group (*n* = 9), 100 mg/kg RSV group (*n* = 9), and 150 mg/kg RSV group (*n* = 9). A murine breast tumor model was established by in situ transplantation of 4T1 breast cancer cell suspensions (100,000 cells) in the right 4th mammary fat pad of female Balb/c mice. Implantations were carried out in a blinded manner, where the researchers performing the injections were blinded to the treatment group. Subsequently, RSV (100 or 150 mg/kg) or an equal volume of vehicle was administered daily via intragastric administration for 35 days, at a consistent time each day to minimize circadian variation. Mice were subsequently euthanized via an overdose of sodium pentobarbital based on their body weight, and their lung tissue was fixed in 4% paraformaldehyde (Sigma Aldrich, Saint Louis, MO, USA) for subsequent research. Animal studies were conducted in accordance with the Ethics Committee of Fujian University of Traditional Chinese Medicine (approval number: FJTCM IACUC 2024018), ensuring that this study complies with the National and Institutional regulations on the care and use of laboratory animals.

### 4.8. HE Staining

We stained according to the instructions in the HE staining kit (Beijing Solarbio Company, Beijing, China). Lung tissues were fixed with 4% paraformaldehyde for 48 h, embedded in paraffin, and sectioned (7 μm sections) onto glass slides. Whole-lung sections that contained the widest cross-sectional area of lung tissue (both left and right lungs) were stained for each mice. Slides were deparaffinized in xylene (2 × 5 min) and rehydrated via an ethanol gradient (100%, 95%, 80%, 70%). Slides were placed in hematoxylin for 10 min, followed by differentiation solution for 10 s, thoroughly rinsed, and then finally stained with eosin stain for 5 min. The stained sections were rapidly dehydrated via an ethanol gradient and mounted with neutral resin. The numbers of nodular aggregates of atypical cells consistent with metastatic carcinoma in both the left and right sections were counted by a blinded investigator.

### 4.9. Statistical Analysis

Experimental data were analyzed using GraphPad Prism software (version 8.0.2). All quantitative results are presented as the mean ± standard deviation (mean ± SD). Prior to statistical comparisons, the normality of the distribution was assessed using the Shapiro–Wilk test, and the homogeneity of variance was verified by the F-test. Intergroup comparisons were performed using Student’s *t*-test (for two groups) or one-way ANOVA with Tukey’s post hoc test (for three or more groups). A *p*-value less than 0.05 was considered statistically significant.

## 5. Conclusions

In summary, the present study confirmed that resveratrol robustly inhibited breast cancer lung metastasis of 4T1 tumor cells by inhibiting the EMT process, which was via its ability to prevent β-catenin nuclear translocation and activation of the Wnt/β-catenin pathway. These findings provide an important experimental basis for the potential clinical use of RSV in inhibiting breast cancer metastasis by targeting the Wnt/β-catenin-regulated EMT process.

## Data Availability

The original contributions presented in this study are included in the article/Supplementary Materials. Further inquiries can be directed to the corresponding authors.

## Supplementary Materials

The following supporting information can be downloaded at: https://www.mdpi.com/article/10.3390/ph19010041/s1, Figure S1: Effect of resveratrol (RSV) on 4T1 cell viability and morphology; Figure S2: Resveratrol (RSV) downregulates N-cadherin expression in vitro and in vivo; Figure S3: Resveratrol (RSV) suppresses Wnt target proteins in vitro and in vivo; Figure S4: Validation of β-catenin knockdown efficiency; Figure S5: Body weight over time and tumor weight at sacrifice in 4T1 tumor-bearing mice.

## Abbreviations

The following abbreviations are used in this manuscript:

RSV	Resveratrol
EMT	Epithelial–mesenchymal transition
FBS	Fetal bovine serum
DMSO	Dimethyl sulfoxide
PEG400	Polyethylene glycol 400
