# Supplementary material for: Resveratrol Prevents Breast Cancer Metastasis by Inhibiting Wnt/β-Catenin Pathway-Mediated Epithelial–Mesenchymal Transition
Source: Pharmaceuticals (Basel). 2025 Dec 23;19(1):41. doi: 10.3390/ph19010041 (PMC12845171; doi:10.3390/ph19010041)
Supplement: Supplementary file 1 [file pharmaceuticals-19-00041-s001.zip › pharmaceuticals-4016407-supplementary.pdf]

Supplementary Figures:

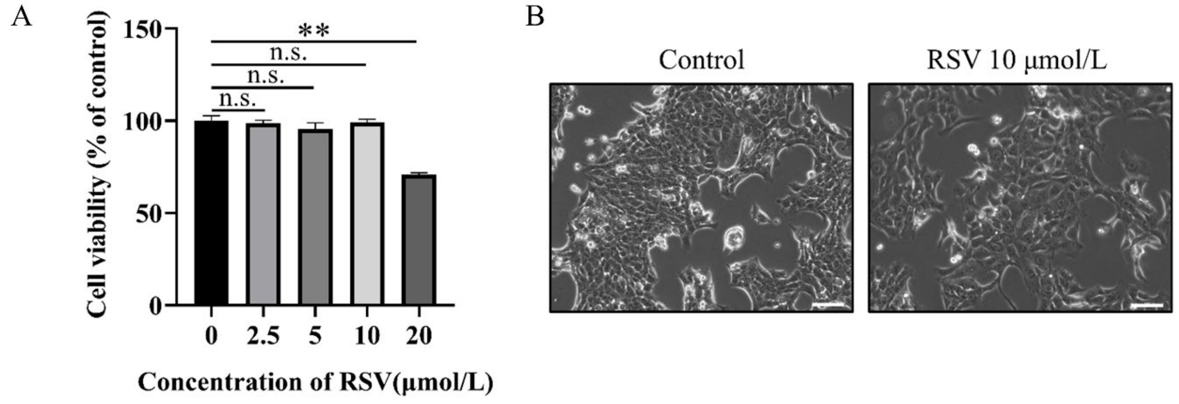

Supplemental Figure S1: Effect of resveratrol (RSV) on 4T1 cell viability and morphology. (A) LDH assay of cell viability in 4T1 cells treated with different concentrations of RSV for 48 h. (B) Representative phase-contrast images of 4T1 cells treated with vehicle or 10 μmol/L RSV for 48 hours (100× magnification). Scale bar, 100 μm. n = 3 independent experiments. Data are presented as mean ± SD. One-way ANOVA, Tukey's post hoc test, not significant (n.s.), **p < 0.01, n = 3 independent experiments.

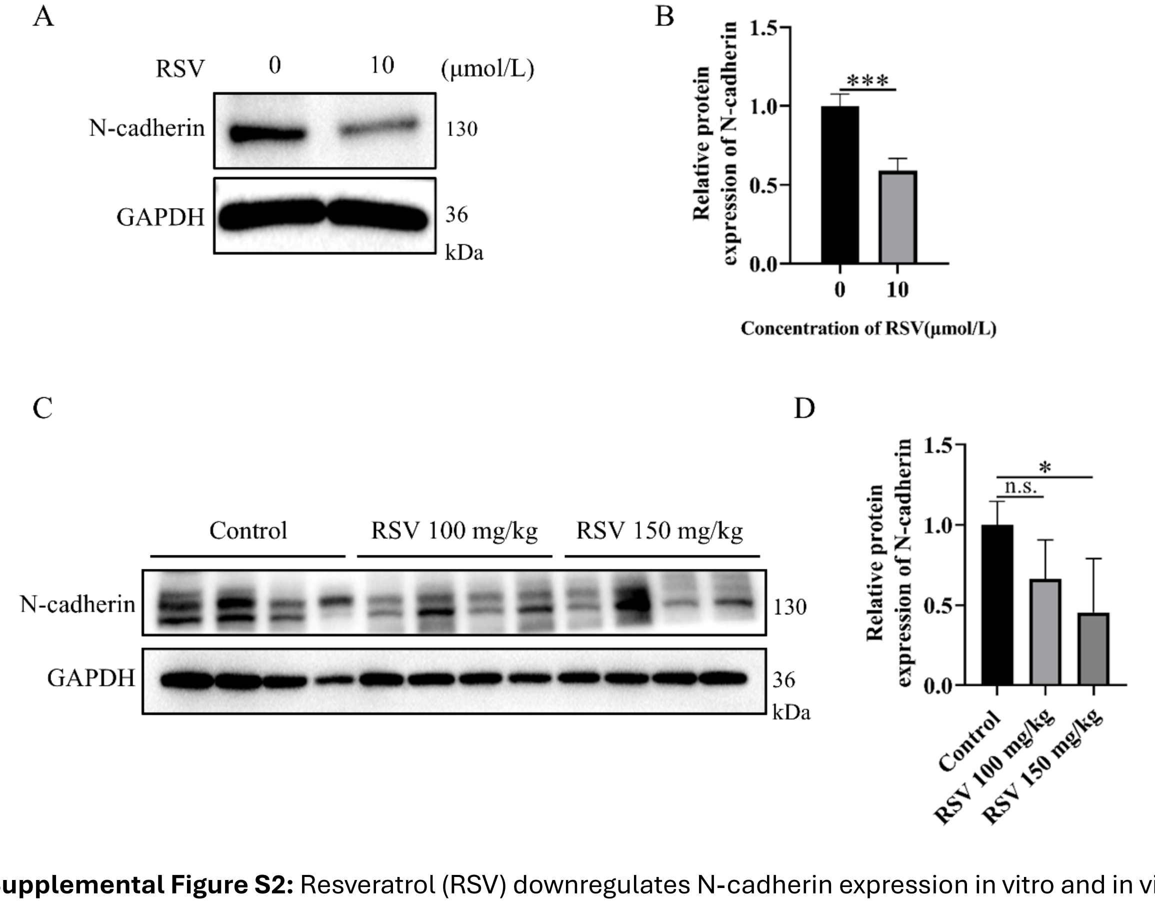

Supplemental Figure S2: Resveratrol (RSV) downregulates N-cadherin expression in vitro and in vivo. Representative Immunoblots (A) and quantification (B) of N-cadherin expression in 4T1 cells treated with RSV for 48 h, n = 4 independent experiments. Mice were administered daily with RSV (100 or 150 mg/kg) or an equal volume of vehicle for 5 weeks. Representative immunoblots (C) and quantification (D) of N-cadherin expression in tumor tissues of mice administrated with the vehicle or RSV for 5 weeks. Data are presented as mean ± SD. One-way ANOVA, Tukey's post hoc test, not significant (n.s.), *p < 0.05, ***p < 0.001, n = 4.

Supplemental Figure S3

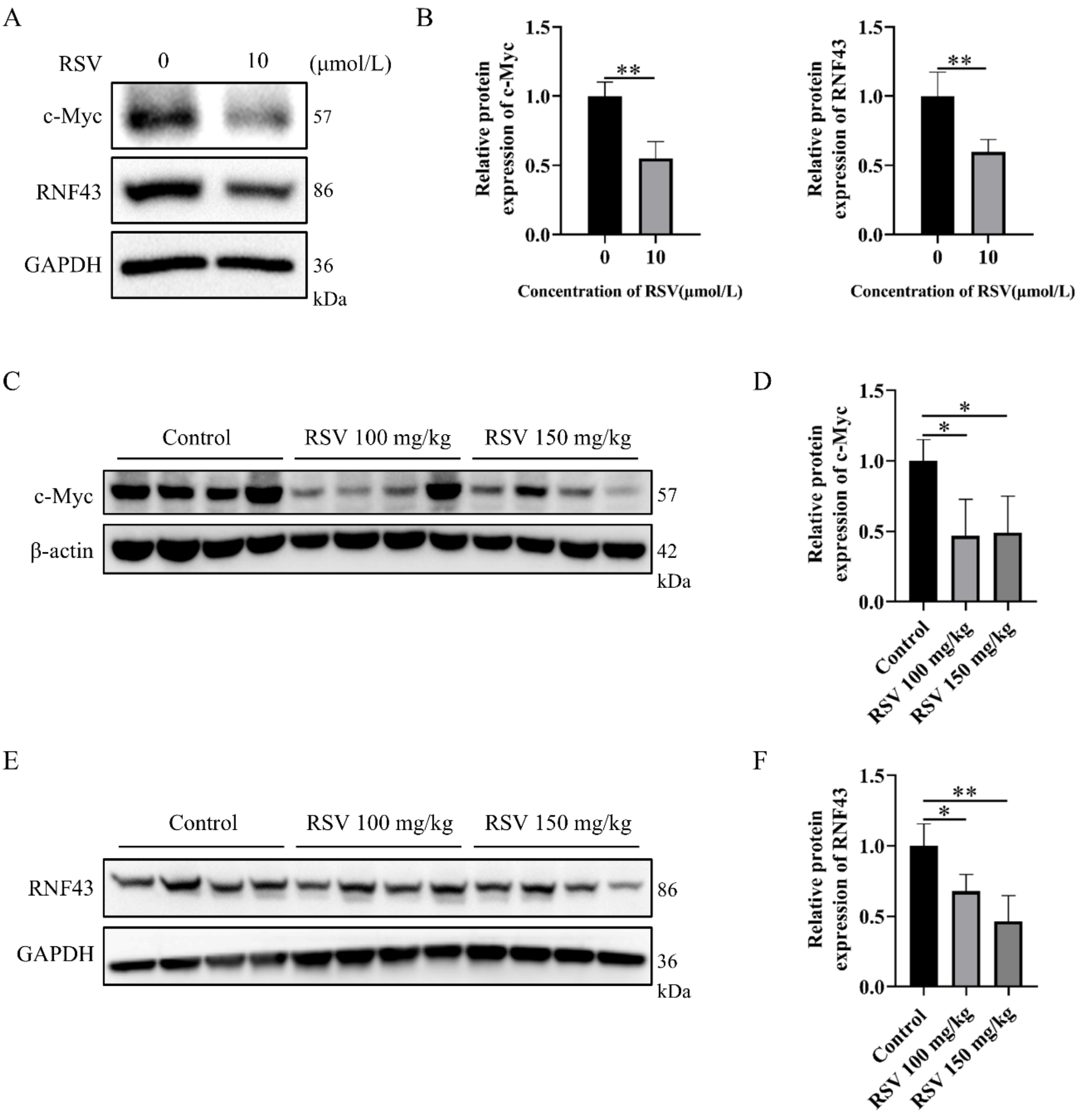

Supplemental Figure S3: Resveratrol (RSV) suppresses Wnt target proteins in vitro and in vivo. Representative Immunoblots (A) and quantification (B) of Wnt target proteins including c-Myc and Rnf43 in 4T1 cells treated with RSV for 48 h, n = 4 independent experiments. Mice were administered daily with RSV (100 or 150 mg/kg) or an equal volume of vehicle for 5 weeks. (C-F) Representative immunoblots (C,E) and quantification (D,F) of Wnt target proteins including c-Myc and Rnf43 in tumor tissues of mice administrated with the vehicle or RSV for 5 weeks. Data are presented as mean ± SD. One-way ANOVA, Tukey's post hoc test. *$p < 0.05$, **$p < 0.01$, n = 4.

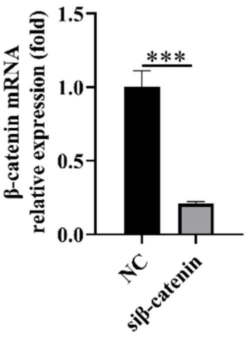

Supplemental Figure S4: Validation of β-catenin knockdown efficiency. Real-time PCR examining the siRNA knockdown efficiency. Relative β-catenin mRNA expression following siRNA knockdown of β-catenin. NC, negative control. Data are presented as mean ± SD. Student's t-test, ***p < 0.001, n = 3 independent experiments.

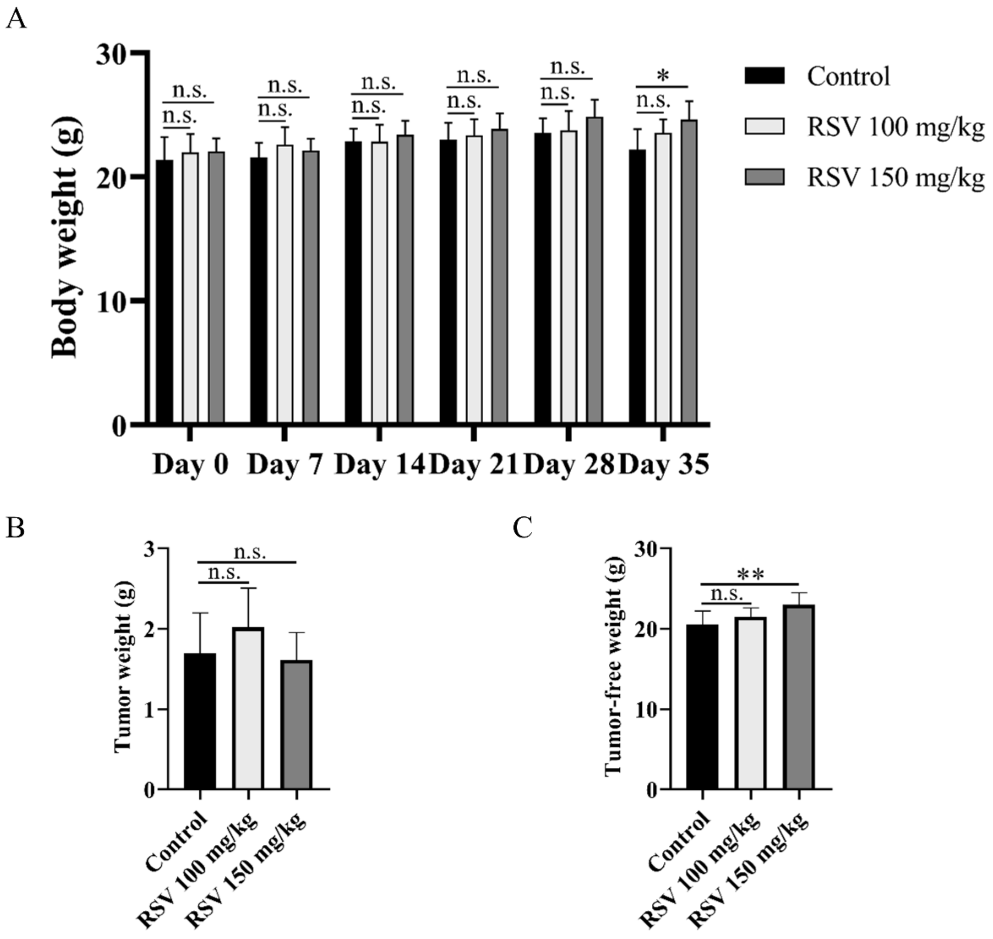

Supplemental Figure S5: Body weight over time and tumor weight at sacrifice in 4T1 tumor-bearing mice. Mice were administered daily with RSV (100 or 150 mg/kg) or an equal volume of vehicle for 5 weeks. (A) Body weight changes in mice every 7 days following 4T1 implantation and daily administration of RSV for 5 weeks. (B) Tumor weight and (C) tumor-free weight of mice measured at sacrifice on day 35. Data are presented as mean ± SD. One-way ANOVA, Tukey's post hoc test, not significant (n.s.), *p < 0.05, **p < 0.01, n = 9 mice in each group.
